# Autoinducer-2 promotes the colonization of *Lactobacillus rhamnosus GG* to improve the intestinal barrier function in a neonatal mouse model of antibiotic-induced intestinal dysbiosis

**DOI:** 10.1186/s12967-024-04991-5

**Published:** 2024-02-18

**Authors:** Riqiang Hu, Ting Yang, Qing Ai, Yuan Shi, Yanchun Ji, Qian Sun, Bei Tong, Jie Chen, Zhengli Wang

**Affiliations:** 1 Children Nutrition Research Center, Chongqing Key Laboratory of Child Neurodevelopmental and Cognitive Disorders, Ministry of Education Key Laboratory of Child Development and Disorders, Children’s Hospital of Chongqing Medical University, National Clinical Research Center for Child Health and Disorders, Chongqing, China; 2 Department of Neonatology, National Clinical Research Center for Child Health and Disorders, Children’s Hospital of Chongqing Medical University, Chongqing, China; 3 Jiangxi Hospital Affiliated Children’s Hospital of Chongqing Medical University, Chongqing, China

**Keywords:** Autoinducer-2, *Lactobacillus rhamnosus GG*, Intestinal barrier function, Antibiotics-induced intestinal flora, Tight junctions, Butyric acid, Hydroxycarboxylic acid receptor 2, Biofilm

## Abstract

**Background:**

Human health is seriously threatened by antibiotic-induced intestinal disorders. Herein, we aimed to determine the effects of Autoinducer-2 (AI-2) combined with *Lactobacillus rhamnosus* GG (*LGG*) on the intestinal barrier function of antibiotic-induced intestinal dysbiosis neonatal mice.

**Methods:**

An antibiotic-induced intestinal dysbiosis neonatal mouse model was created using antibiotic cocktails, and the model mice were randomized into the control, AI-2, *LGG*, and *LGG* + AI-2 groups. Intestinal short-chain fatty acids and AI-2 concentrations were detected by mass spectrometry and chemiluminescence, respectively. The community composition of the gut microbiota was analyzed using 16S rDNA sequencing, and biofilm thickness and bacterial adhesion in the colon were assessed using scanning electron microscopy. Transcriptome RNA sequencing of intestinal tissues was performed, and the mRNA and protein levels of HCAR2 (hydroxycarboxylic acid receptor 2), claudin3, and claudin4 in intestinal tissues were determined using quantitative real-time reverse transcription PCR and western blotting. The levels of inflammatory factors in intestinal tissues were evaluated using enzyme-linked immunosorbent assays (ELISAs). D-ribose, an inhibitor of AI-2, was used to treat Caco-2 cells in vitro.

**Results:**

Compared with the control, AI-2, and *LGG* groups, the *LGG* + AI-2 group showed increased levels of intestinal AI-2 and proportions of *Firmicutes* and *Lacticaseibacillus*, but a reduced fraction of *Proteobacteria*. Specifically, the *LGG* + AI-2 group had considerably more biofilms and *LGG* on the colon surface than those of other three groups. Meanwhile, the combination of AI-2 and *LGG* markedly increased the concentration of butyric acid and promoted Hcar2, claudin3 and claudin4 expression levels compared with supplementation with *LGG* or AI-2 alone. The ELISAs revealed a significantly higher tumor necrosis factor alpha (TNF-α) level in the control group than in the *LGG* and *LGG* + AI-2 groups, whereas the interleukin 10 (IL-10) level was significantly higher in the *LGG* + AI-2 group than in the other three groups. In vitro, D-ribose treatment dramatically suppressed the increased levels of Hcar2, claudin3, and claudin4 in Caco-2 cells induced by AI-2 + *LGG*.

**Conclusions:**

AI-2 promotes the colonization of *LGG* and biofilm formation to improve intestinal barrier function in an antibiotic-induced intestinal dysbiosis neonatal mouse model.

**Supplementary Information:**

The online version contains supplementary material available at 10.1186/s12967-024-04991-5.

## Background

Antibiotic-induced intestinal disorders have become a serious threat to human health [1]. Antibiotics disrupt the composition of the gut microbiota during the early life and development of the immune system, leading to long-term diseases such as obesity, asthma, and allergic diseases [2–4]. *Lactobacillus*, an important component of the intestinal flora, plays a vital role in maintaining intestinal stability [5], including the composition of the intestinal microbiota, the intestinal barrier, immune function, competitive adhesion with virulence factors of pathogens, and the secretion of biologically active anti-infective compounds [6]. Unfortunately, antibiotics can decrease the number of *Lactobacillus* [7], which aggravates the severity of intestinal infections, irritable bowel syndrome, and inflammatory bowel disease [8–10]. Therefore, it is crucial to promote the growth and colonization of *Lactobacillus* to maintain a balance that could promote the intestinal health.

Autoinducer-2 (AI-2) is a novel autoinducer produced by the bacterial *luxS* gene [11], which acts as a universal regulator of bacterial gene expression in a feedback manner [12]. AI-2 plays a crucial role in the intestines [13], where it contributes to the adhesion and colonization properties of various probiotics, such as *Lactobacillus acidophilus*, *Lactobacillus plantarum*, and *Bifidobacterium bifidum* [14–17], and modulates intestinal immune function [18]. Studies have shown that exogenous AI-2 reshaped the gut microbiota and alleviated intestinal inflammation in a neonatal mouse model of necrotizing enterocolitis [19, 20]. Nevertheless, the detailed mechanisms remain to be determined.

*Lactobacillus rhamnosus GG (LGG*) possesses unique characteristics that allow it to thrive in digestive acid pH and bile-containing environments, as well as attaching to enterocytes in intestinal epithelial cells [21]. These abilities enable *LGG* to successfully colonize the intestine, making it an effective probiotic option [22]. A recent study reported that the colonization of *LGG* is influenced by AI-2 [23]. However, the effect of exogenous AI-2 on the colonization of *LGG* in neonatal mice with antibiotic-induced intestinal dysbiosis has not been investigated. Therefore, we hypothesized that exogenous AI-2 would promote the colonization of *LGG*, correct microbiota dysbiosis, and improve the intestinal barrier function.

In this study, the 16S rDNA gene sequences of gut microbiota from neonatal mouse with antibiotic-induced intestinal dysbiosis were analyzed and then biofilm thickness and bacterial adhesion in the colon were assessed using scanning electron microscopy (SEM). Next, transcriptome sequencing of intestinal tissues was performed to identify critical genes associated with intestinal barrier function. Finally, an inhibitor of AI-2 was used in Caco-2 cells (immortalized human colon cells) to confirm the in vitro effects of AI-2 combined with *LGG* on gut barrier-associated proteins.

## Methods

### Animal experiments

C57BL/6 mice were obtained from Chongqing Medical University and housed in specific pathogen free (SPF) conditions. Female and male mice were caged together at a 1:2 ratio to obtain pregnant mice. SPF neonatal C57BL/6 J pups were given antibiotics via gavage on postnatal day 3. Antibiotics were administered at 09:00 am daily for 4 consecutive days of treatment at 0.03 mL/g/day. The antibiotic cocktails were: ampicillin sodium (25 mg/kg), metronidazole (50 mg/kg), piperacillin sodium tazobactam sodium (100 mg/kg), and vancomycin (10 mg/kg) (all Meilunbio, Dalian, China) [24, 25].

### Preparation of *LGG*

*Lactobacillus rhamnosus GG* was grown as described previously [26]. In brief, *Lactobacillus rhamnosus GG* (BNCC136673, China) were anaerobically incubated in De Man, Rogosa, and Sharpe (MRS) medium (Hopebio, Qingdao, China) at 37 °C for 24 h for activation. The OD600 of activated LGG was adjusted to 0.5, corresponding to approximately ~ 10^8^ colony forming units (CFU)/ml, and then centrifuged at 5000 rpm for 5 min at 4 °C, washed and resuspended twice in sterile saline before use.

### *LGG* and AI-2 solution gavage

After antibiotic gavage, 7-day-old neonatal mice were randomly divided into the control, AI-2, *LGG*, and *LGG* + AI-2 groups, in which the control and AI-2 groups were gavaged with 50 μL of saline or AI-2 (500 nmol/L) solution 5 times per day; the *LGG* group received 50 μL of *LGG* solution (containing 1 × 10^8^ CFU), or 50 μL (containing both AI-2 and *LGG*) mixed solution once per day; at the remaining four time points they were treated with 50 μL of saline (*LGG* group) and 50 μL of AI-2 (500 nmol/L; *LGG* + AI-2 group) solution for 3 consecutive days.

### Histological observation

The four groups of pups were decapitated on postnatal day 10. Histological examination was performed as previously described [27]. In brief, intestines were removed from the body of each mouse, and a 1 cm distal part of the ileum was fixed in paraformaldehyde solution (4%). Then, the sample was dehydrated and embedded in paraffin. Next, the sample was cut into 4-mm slices. Subsequently, the 4-mm tissue slices were stained with hematoxylin and eosin (HE). ImageJ software (NIH, Bethesda, MD, USA) was used to measure the length of the intestinal tissue villi in each group to assess the intestinal development and the degree of injury [28].

### Detection of AI-2 activity and *LGG* in mouse intestines

The method was carried out as previously described [29]. In brief, the colon of each mouse was flushed with 400 µL of 2216E medium (Hopebio, Qingdao, China). The fecal filtrates were collected in sterile tubes, vortexed, and centrifuged. *Lactobacillus rhamnosus GG* (*LGG*) was cultured twice in MRS broth, incubated at 37 °C for 16 h, washed twice with saline, re-suspended in 0.7 mL of fresh MRS broth at 1 × 10^8^ CFU, and 0.2 mL of AI-2 (500 nmol/L) was added to form a mixture of *LGG* + AI-2, and then 0.1 mL aliquots of different concentrations of D-ribose were added, respectively, so that the final 1 mL mixture contained 0, 10, 50, or 100 mmol/L D-ribose, which was then incubated for 3,6, 9, 16, 24, and 30 h. The bacterial supernatant was collected by centrifugation at 12,000 × *g* for 10 min at 4 °C. The sterile supernatants were obtained by filtration through a 0.22 µm filter (Millipore, Billerica, MA, USA). (S)-4,5-dihydroxypentane-2,3-dione (DPD) (as the AI-2 precursor) was sourced from OmmScientific (Dallas, TX, USA). The AI-2 levels in the samples were assayed using the *Vibrio harveyi* BB170 reporter. BB170 was cultured in 2216E medium (Hopebio) at 30 °C for 18 h and then diluted 1:5000 in fresh 2216E. For the AI-2 test, the samples were combined with the *V. harveyi* BB170 dilutions. Additionally, 20 µL of 1 mM AI-2 (as a positive control), fecal filtrate, bacterial supernatant, and 2216E medium (as a negative control) were pipetted into a 96-well plate (Corning Inc. Corning, NY, USA). Then, 180 µL of BB170 dilution was added to each well to make a total of 200 µL and the plate was agitated at 30 °C and 120 rpm. After 30 min of incubation, the luminescence of the samples was recorded using a multimode microplate reader (BioTek Instruments, Synergy H1, Winooski, VT, USA) at intervals of 30 min until the signal was lowest in the negative control.

### Fecal microbiota analysis

Total microbial genomic DNA was extracted using a QIAamp FAST DNA Stool Mini-Kit (Qiagen, Hilden, Germany) according to manufacturer’s instructions. The quality and concentration of the DNA were determined using 1.0% agarose gel electrophoresis and a NanoDrop^®^ ND-2000 spectrophotometer (Thermo Scientific Inc., Waltham, MA, USA) and kept at − 80 ℃ before further use. The hypervariable region V3-V4 of the bacterial 16S rRNA gene was amplified using primer pair 338F (5ʹ-ACTCCTACGGGAGGCAGCAG-3ʹ) and 806R (5ʹ-GGACTACHVGGGTWTCTAAT-3ʹ) [30] in an ABI GeneAmp^®^ 9700 PCR thermocycler (ABI, Foster City, CA, USA). The amplification procedure was as described previously^20^. The PCR product was extracted from a 2% agarose gel, purified, and then quantified using a Quantus^™^ Fluorometer (Promega, Madison, WI, USA). Novaseq sequencer (Illumina, San Diego, USA) was used to carry out 2 × 250 bp paired-end sequencing, and the corresponding reagent is NovaSeq 6000 SP Reagent Kit (Illumina, San Diego, USA). Bioinformatic analysis of the gut microbiota was carried out using the Majorbio Cloud platform (https://cloud.majorbio.com). Based on the ASVs information, the community richness indicators Ace, Chao, Simpson, Sobs, and Shannon index were calculated with Mothur v1.30.2 [31]. The similarity among the microbial communities in different samples was determined by principal coordinate analysis (PCoA) based on Bray–curtis dissimilarity using Vegan v2.4.3 package.

### Scanning electron microscopy of *LGG* and mouse colonic tissue

The OD_600_ of the activated *LGG* culture was adjusted to 0.8, and 1.5 mL of the culture was poured into a 2 mL EP tube and centrifuged at 10,000 × *g* for 15 min at 4 ℃. The supernatant was discarded, and 4% glutaraldehyde was gently added along the tube wall, and the tube was stored at 4 °C. Colon tissues were isolated and rinsed twice with 4 °C phosphate-buffered saline (PBS), and then fixed in 4% glutaraldehyde at 4 °C until they were transported to the electron microscopy room of Chongqing Medical University. Samples were then washed and sequentially dehydrated with increasing concentrations of ethanol (30, 50, 70, 90, and 100%) for 35 min and soaked in pure tert-butanol. The samples were freeze-dried in a vacuum freeze dryer (Ningbo Scientz Biotechnology Co. Ltd., Ningbo, China). After drying, the samples were mounted on holders, gold coated using an MC1000 sputter coater (Hitachi, Tokyo, Japan), and examined by SEM (SU8010, Hitachi).

### Intestinal tissue transcriptome

Total RNA was extracted from the tissue using the QIAzolLysisReagent according the manufacturer’s instructions (Qiagen, Germany). Then RNA quality was determined using a 5300 Bioanalyser (Agilent, Santa Clara, CA, USA) and quantified using an ND-2000 (NanoDrop Technologies, Wilmington, ME, USA). Only high-quality RNA samples (OD260/280 = 1.8 ~ 2.2, OD260/230 ≥ 2.0, RNA integrity number (RIN) ≥ 6.5, 28S:18S ≥ 1.0, and > 1 μg) were used to construct the sequencing library. RNA purification, reverse transcription, library construction, and sequencing were performed at Majorbio Bio-pharm Biotechnology Co., Ltd. according to the manufacturer’s instructions (Illumina). Differential expression analysis was performed using the DESeq2 [32]. DEGs with |log2FC| ≧0.58 and P value ≤ 0.05 (DESeq2) were considered to be significantly different expressed genes. In addition, functional-enrichment analysis including Gene Ontology (GO) was performed to identify which DEGs were significantly enriched in GO terms and metabolic pathways at Bonferroni-corrected P-value ≤ 0.05 compared with the whole-transcriptome background (GO, http://www.geneontology.org, p < 0.05).

### Western blotting

The intestinal tissues and Caco-2 cells were homogenized and lysed using radioimmunoprecipitation assay (RIPA) lysis buffer (keygen BioTECH, Nanjing, China), supplemented with 1% phenylmethylsulfonyl fluoride (PMSF) (keygen BioTECH). The concentration of the extracted proteins was determined using the Bicinchoninic acid assay kit (keygen BioTECH). Protein solutions were mixed with sodium dodecyl sulfate (SDS) sample buffer (keygen BioTECH) in a 4:1 ratio and denatured in boiling water for 10 min. Protein samples were separated on a 10% polyacrylamide gel and then transferred onto a polyvinylidene difluoride (PVDF) membrane (Millipore). The membrane was blocked in rapid blocking solution at room temperature for 10 min, followed by overnight incubation at 4 °C with antibodies against hydroxycarboxylic acid receptor 2 (Hcar2) (Affinity Biosciences, Jiangsu, China), claudin3 (Affinity Biosciences), claudin4 (ZENBIO Biotechnology, Chengdu, China), β-actin (ABclonal, Wuhan, China), and β-Tubulin (ZENBIO Biotechnology). Subsequently, the membrane was incubated with a labeled secondary anti-rabbit antibody for 1 h, and the immunoreactive protein bands were detected using an enhanced chemiluminescence (ECL) kit (Millipore) and visualized using the Bio-Rad ChemiDoc™ Touch Imaging System (Bio-Rad, Hercules, CA, USA). Image analysis was performed using Image Lab and ImageJ software.

### Enzyme linked immunosorbent assay (ELISA)

The levels of tumor necrosis factor alpha (TNF-α) and interleukin 10 (IL-10) in intestinal tissue supernatants were determined using commercially available kits (Mouse TNF-α and IL-10 ELISA Kits; Neobioscience, Shenzhen, China) according to the manufacturer's instructions.

### Quantitative real-time reverse transcription PCR of intestinal tissue and caco-2 cells

RNA from mouse intestinal tissues and Caco-2 cells was extracted using an RNA extraction kit (Promega, Beijing, China). The RNA was then reverse transcribed into cDNA. The quantitative real-time PCR (qPCR) step was performed using the CFX96 real-time PCR detection system (Bio-Rad). *ACTB* or *Actb* (encoding beta-actin) was used as the housekeeping gene, and the mRNA levels of *Hcar2/HCAR2*, *Cldn3/CLDN3* (claudin3), and *Cldn4/CLDN4* (claudin4) were normalized against it. The following primer sequences were used: *Actb* (mouse) forward, 5ʹ-ACTGCCGCATCCTCTTCCTC-3ʹ; *Actb* (mouse) reverse, 5ʹ-AACCGCTCGTTGCCAATAGTG-3ʹ; *Hcar2* (mouse) forward, 5ʹ- CCTGACTGTCCACCTCCTCTATAC-3ʹ; *Hcar2* (mouse) reverse, 5ʹ- ATCGTGCCACCTGAAGTTGTAAC-3ʹ; *Cldn3* (mouse) forward, 5ʹ-GCCAACACCATCATCAGGGATTTC-3ʹ; *Cldn3* (mouse) reverse, 5ʹ-GCAGGAGCAACACAGCAAGG-3ʹ’; *Cldn4* (mouse) forward, 5ʹ- GGATGCTTCTCTCAGTGGTAGGG-3ʹ; *Cldn4* (mouse) reverse, 5ʹ- AGGACACGGGCACCATAATCAG-3ʹ; *ACTB* (human) forward, 5ʹ- CCACGAAACTACCTTCAACTCCATC-3ʹ; *ACTB* (human) reverse, 5ʹ- AGTGATCTCCTTCTGCATCCTGTC-3ʹ; *HCA2* (human) forward, 5ʹ- CGGACAGCAGCCATCATCTCTTG-3ʹ; *HCAR2* (human) reverse, 5ʹ- GCATCTTCTTCTTCAGGAGGTGGAC-3ʹ; *CLDN3* (human) forward, 5ʹ-TTCATCGGCAGCAACATCATCAC-3ʹ; *CLDN3* (human) reverse, 5ʹ- AGCAGCGAGTCGTACACCTTG-3ʹ; *CLDN4* (human) forward, 5ʹ- ATCGGCAGCAACATTGTCACCTC-3'; *CLDN4* (human) reverse, 5ʹ’- CAGCAGCGAGTCGTACACCTTG-3ʹ. The results are presented as 2^−ΔΔCt^ ± SEM.

### Detection of short-chain fatty acids (SCFAs)

Solid samples (25 mg) were accurately weighed into 2 mL grinding tubes and added with 500 µL water containing 0.5% phosphoric acid. The samples were frozen and ground at 50 Hz for 3 min, repeated twice, followed by ultrasound treatment for 10 min, and centrifugation at 4 °C and 13000 × *g* for 15 min. Then, 200 μL of the supernatant was removed into a 1.5 mL centrifuge tube, and added with 0.2 mL N-butanol solvent containing internal standard 2-ethylbutyric acid (10 μg/mL) for exaction. Finally, the sample was vortexed for 10 s, treated with ultrasound at low temperature for 10 min, followed by centrifugation at 4 °C and 13000 × *g* for 5 min, after which the supernatant was carefully transferred to a sample vial for gas chromatography–mass spectrometry analysis (Shanghai Meiji Biomedical Technology Co., Ltd, Shanghai, China).

### Effect of AI-2 + *LGG* and D-ribose co-culture on caco-2 cells

Caco-2 cells were inoculated at 2 × 10^5^ cells/well into 6 cm dishes (Corning) and cultured in an incubator at 37 °C, 5% CO_2_, and 95% air until a monolayer cell model was formed. *Lactobacillus rhamnosus (LGG)* was cultured in MRS broth at 37 °C for 16 h. It was washed twice with saline and resuspended in 0.7 mL of antibiotic-free Dulbecco’s modified Eagle’s medium (DMEM) at 1 × 10^8^ CFU. Then, 0.2 mL of AI-2 (500 nmol/L) was added to form the *LGG* + AI-2 mixture. Next, 0.1 mL of 500 mmol/L D-ribose was added for the *LGG* + AI-2 + D-Ribose group; 0.1 ml of saline was added for the *LGG* + AI-2 group; 0.9 mL of antibiotic-free DMEM with 0.1 mL of D-ribose (500 mmol/L) was added for the D-ribose group; and 1 mL of DMEM without added antibiotics was added for the control group. All the mixtures were spread on separate cell monolayers, followed by incubation at 37 °C for 6 h. The cell monolayers were washed with pre-cooled saline twice to extract cellular proteins and RNA.

### Adhesion assay to caco-2 cells

Caco-2 cell culture and adhesion assays were performed as previously described with minor modifications [33–35]. Caco-2 cells were inoculated into 12-well plates at a concentration of 2*10^5^ cells/well and cultured in DMEM medium containing 10% fetal bovine serum until monolayers were formed, and 0.9% saline was rinsed twice. The aforementioned LGG was resuspended in DMEM without added antibiotics at a concentration of 1*10^8^ CFU/mL. 12-well plates were added containing 1 ml of bacterial resuspension and 0.25 ml of sterile saline for the AI-2 0 nm group; containing 1 ml of bacterial resuspension and 0.25 ml of 50 nmol/L AI-2 for the 10 nm group; with 1 ml of bacterial resuspension and 0.25 ml of 500 nmol/L AI-2 for the 100 nm group; with 1 ml of bacterial resuspension and 0.25 ml of 2500 nmol/L AI-2 500 nm group [1]. Plate counting method: After 3 h of co-incubation at 37 °C, 5% CO2 incubator, 0.9% saline rinsed three times to remove the bacterial suspension and unadhered bacteria, and then the cells were digested with 0.2 ml of trypsin, and 0.3 ml of DMEM medium with 10% fetal bovine serum was used after the cells were detached, and the adhering bacteria were collected. The mixtures were diluted 1000 in saline and 50ul of the diluted mixture was taken and inoculated on MRS agar to assess the number of adherent bacteria surviving. The adhesion of the strains was expressed as the adhesion rate, which was calculated according to the following formula: adhesion rate (%) = lg CFU Nt/lg CFU N0 × 100% (N0 is the number of LGG before treatment and Nt is the number of bacteria after treatment). (2) Microscopic observation: The procedure was the same as the Caco-2 adhesion experiment described above. After co-incubation for 3 h at 37 °C, 5% CO2 incubator, 0.9% saline was rinsed three times to remove unadhered bacteria. The adherent bacteria were fixed with 4% paraformaldehyde for 15 min and Gram stained. The adherent bacteria were counted under a microscope to observe the adhesion.

### Quantitative real-time PCR of LGG

Lactobacillus rhamnosus (LGG) was cultured twice in MRS broth and incubated at 37 °C for 16 h. The culture was then washed twice with saline and re-suspended in MRS broth at a concentration of 1 × 10^8^ CFU/mL. In 96-well plates, 160 μL of the bacterial resuspension was added, along with 40 μL of 2500 nmol/L AI-2 for the 500 nm group; 160 μL of the bacterial resuspension and 40 μL of 500 nmol/L AI-2 for the 100 nm group; 160 μL of the bacterial resuspension and 40 μL of 50 nmol/L AI-2 for the 10 nm group; and 160 μL of the bacterial resuspension and 40 μL of sterile saline for the AI-2 0 nm group. The plates were then incubated for a total of 6 h at 37 °C in a 5% CO2 incubator. The bacteria in the 96 wells were collected by centrifugation at 8000 rpm for 5 min at 4 °C. Total bacterial RNA was extracted using a previously described method [36]. RT-PCR analysis was performed using the CFX96 real-time PCR detection system (Bio-Rad, USA). GAPDH was used as a housekeeping gene and mRNA levels of spaC, pili, and Pilus444 were normalized to it. Primer sequences were used as described previously [37]: *GAPDH* (LGG_00933) forward, 5ʹ- GATCGTTTCTGCAGGTTCTT -3ʹ; *GAPDH* (LGG_00933) reverse, 5ʹ- CCGTTCAATTCTGGGATAAC -3ʹ; *spaC* (LRHM_0428) forward, 5ʹ- CAACTTGATGGGACAACGTA -3ʹ; *spaC* (LRHM_0428) reverse, 5ʹ- TCTGGTGCTTTTGTTTCTGA -3ʹ; *pili* (LGG_02339) forward, 5ʹ- GATTATCGGGTTGATTCTGG-3ʹ; *pili* (LGG_02339) reverse, 5ʹ- AAATCGCCTTCGTACATCTC-3ʹ; *Pilus444* (LGG_00444) forward, 5ʹ- CAACTTGATGGGACAACGTA-3ʹ; *Pilus444* (LGG_00444) reverse, 5ʹ- TTTGCAGGATTGCTTTGATA-3ʹ’;. Results are expressed as 2^-ΔΔCt ± SEM.

### Statistical analysis

Data are presented as mean ± standard error of the mean (SEM), the median, or minimum and maximum values according to the normality of the distribution. Cohen classified effect size as small (d = 0.2), medium (d = 0.5) and large (d = 0.8) [38]. One-way analysis of variance (ANOVA), Duncan's test, tests for normality, and a logarithmic test (D') were performed using SPSS 22 (IBM Corp., Armonk, NY, USA) and GraphPad Prism 9 (GraphPad Software, San Diego, CA, USA). Statistical differences were defined as significant at P < 0.05.

## Results

### AI-2 combined with *LGG* elevated the intestinal AI-2 concentration in the antibiotic-induced intestinal dysbiosis neonatal mouse model

**Fig. 1:**
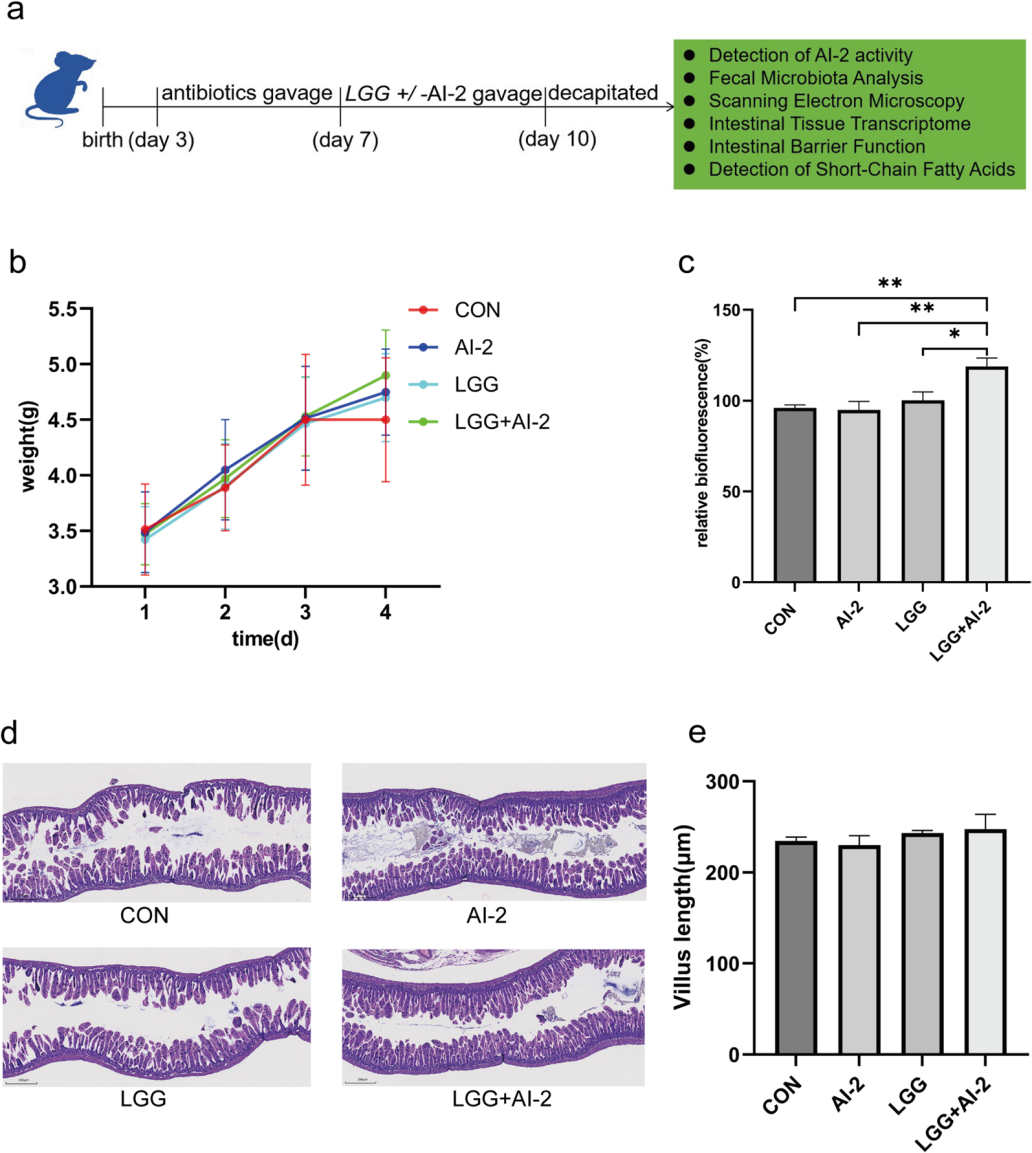
Effect of AI-2 with *LGG* on the intestines of the antibiotic-induced neonatal mouse model. **a **The experimental procedure of the study. The variations in **b** body weight (n = 8 per group), **c** intestinal AI-2 concentration (n = 7 per group), **d** pathological morphology of ileal intestines (n = 3 per group). **e** Quantification of intestinal villus length (n = 3 per group). Values are presented as the mean± SEM. Significance was tested using one-way ANOVA. AI-2, autoinducer-2; *LGG*, *Lactobacillus rhamnosus GG*. ^*^P < 0.05, ^**^P < 0.01

The experimental procedure is outlined in Fig. 1a. On the fourth day, the *LGG* + AI-2 group of mice outweighed the other three groups, while the control group exhibited the opposite trend. However, the differences did not demonstrate statistical significance (Fig. 1b). The *LGG* + AI-2 group of mice had a substantially greater intestinal AI-2 concentration compared with that in the control group, AI-2 group, and *LGG* group (Fig. 1c, P < 0.01, *P* < 0.01, and *P* < 0.05, respectively). However, the pathological changes and villus length of the ileal intestine (Fig. 1d, e) did not differ significantly among the four groups, and other general conditions such as mortality, vitality, hair luster, subcutaneous fat, bloating, diarrhea, and gastric retention also did not differ among the groups.

### The combination of AI-2 with *LGG* elevated the proportions of *Firmicutes* and *Lacticaseibacillus* and decreased the fraction of *Proteobacteria* in the antibiotic-induced intestinal dysbiosis neonatal mouse model

The gut microbiota profile serves as an important regulator of host intestinal homeostasis and the immune system [39]. Studies have shown that the mechanical, chemical, and immunological barriers of the gut are controlled in part by the gut bacteria [40]. Using 16S rDNA sequencing, the bacterial community structure was examined to investigate the effects of AI-2 coupled with *LGG* on the intestinal flora in antibiotic-induced intestinal dysbiosis neonatal mouse model. The community richness indicators Ace, Chao, Simpson, Sobs, and Shannon index did not indicate variations among the four groups (Additional file 1: Table S1). At the phylum, class, and order levels, PCoA investigation using the Bray–Curtis model showed that the *LGG* group was considerably separated from the *LGG* + AI-2 group in the direction of the PC1 axis, and PCoA analysis using weighted UniFrac distances confirmed this finding (Additional file 5: Fig S1). These findings showed a significant difference between the *LGG* + AI-2 group and the *LGG* group in the gut microbial community of neonatal mice with antibiotic-induced intestinal dysbiosis.

**Fig. 2:**
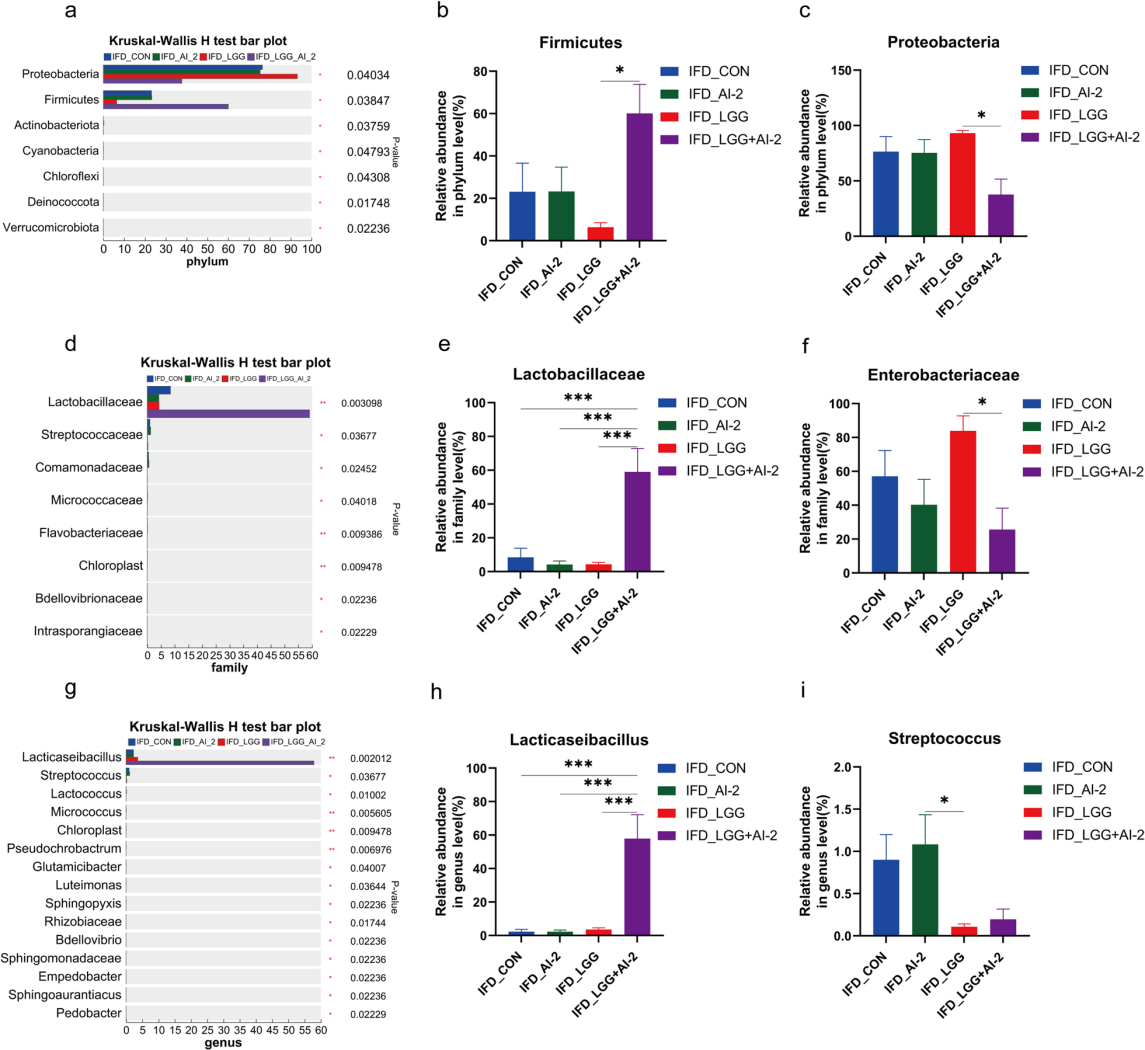
Changes in the gut microbiota composition following AI-2 combined with *LGG* treatment. The differences at the **a**–**c** phylum level, **d**–**f** family level, and **g**–**i** genus level based on Kruskal-Wallis H test. n = 8 per group. Values are presented as the mean ± SEM. Significance was tested using Kruskal-Wallis H test. *P < 0.05, **P < 0.01, ***P < 0.001

Next, the community composition at the phylum, family, and genus levels in the control, AI-2, *LGG*, and *LGG* + AI-2 groups were examined to identify specific alterations in the gut microbiota of antibiotic-induced intestinal dysbiosis neonatal mouse model. Firmicutes (23.13%), Proteobacteria (76.45%), and Bacteroidota (0.15%) were the three dominant phyla in the control group, and Firmicutes (23.25%), Proteobacteria (75.36%), and Bacteroidota (0.17%) were dominant in the AI-2 group. However, Firmicute*s* (6.35%), Proteobacteria (93.21%), and Bacteroidota (0.12) were the most prevalent phyla in the *LGG* group. Notably, the most predominant phyla in the *LGG* + AI-2 group were Firmicutes (60.07%), *Proteobacteria* (37.74%), and Bacteroidota (0.11%) (Additional file 2: Table S2). At the phylum level (Fig. 2a–c), the mean relative abundance of Firmicutes was higher in the *LGG* + AI-2 group than in the *LGG* group (*P* < 0.05, effect size 0.69). However, there was no significant difference (*P* > 0.05) between the AI-2 group and the control group. Similarly, the mean relative abundance of Proteobacteria was lower in the *LGG* + AI-2 group than in the *LGG* group (*P* < 0.05, effect size 0.7), but the difference was not significant (*P* > 0.05) between the AI-2 group and the model control group. As shown in Additional file 3: Table S3 and Additional file 4: Table S4, the *LGG* + AI-2 group had the greatest ratio of *Lactobacillaceae* family and the *Lacticaseibacillus* *spp*. genus (59.08%, 57.85%), followed by the control group (8.43%, 2.28%), the AI-2 group (4.21%, 2.27%), and the *LGG* group (4.28%, 3.57%). At the family level (Fig. 2d–f), the mean relative abundance of *Lactobacillaceae* in the *LGG* + AI-2 group was significantly higher than that of the control (*P* < 0.001, effect size 0.65), AI-2 (*P* < 0.001, effect size 0.7), and *LGG* groups (*P* < 0.001, effect size 0.7), and the mean relative abundance of *Enterobacteriaceae* was dramatically lower in the *LGG* + AI-2 group than in the *LGG* group (*P* < 0.05, effect size 0.68). At the genus level (Fig. 2g–i), the mean relative abundance of *Lacticaseibacillus spp.* in the *LGG* + AI-2 group was substantially greater than that in the control (*P* < 0.001, effect size 0.69), AI-2 (*P* < 0.001, effect size 0.7), and *LGG* groups (*P* < 0.001, effect size 0.69), whereas the *LGG* group exhibited a lower mean relative abundance of *Streptococcus spp.* compared with that in the AI-2 group (*P* < 0.05, effect size 0.57). Moreover, the *LGG* + AI-2 group had a lower mean abundance of *Streptococcus spp.* than the AI-2 and control groups; however, the difference was not significant (*P* > 0.05). These data showed that AI-2 combined with *LGG* elevated the percentage of Firmicutes and *Lacticaseibacillus spp.* and lowered the fraction of *Proteobacteria*, which in turn reshaped the flora structure of antibiotic-induced intestinal dysbiosis neonatal mouse model.

### AI-2 increased the number of *LGG* adhesions and biofilm formation in the antibiotic-induced intestinal dysbiosis neonatal mouse model

**Fig. 3:**
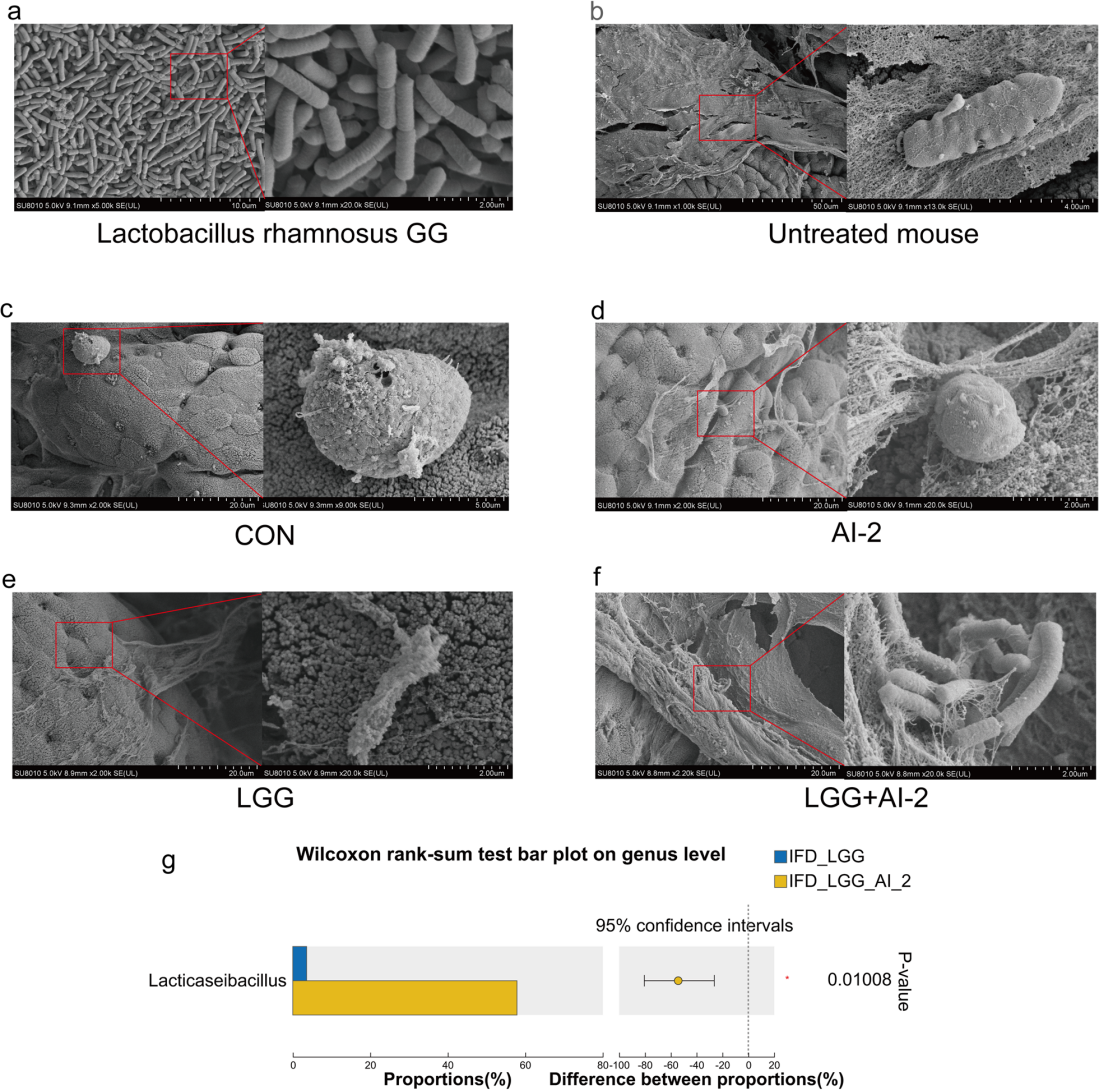
Observation of the adhesion of *LGG* on the surface of intestinal mucosa by SEM. **a** Morphological observation of *LGG* under a scanning electron microscope. Changes to biofilms and bacterial adhesion in the colon of **b** untreated mouse, **c** control, **d** AI-2, **e** *LGG*, **f** *LGG*+AI-2 groups (n = 3 per group) under the scanning electron microscope. The differences at the **g** genus level between *LGG* group and *LGG*+AI-2 group (n = 8 per group). Values are presented as the mean ± SEM. Significance was tested using Wilcoxon rank-sum test

Colonic scanning electron microscopy and fecal flora analyses were used to examine the potential effect of AI-2 on intestinal *LGG*. Under scanning electron microscopy, *LGG* was typically 0.5–0.6 × 1.6–2.0 μm in size, and the organisms were often elongated, rounded rods without flagella (Fig. 3a). Additionally, we observed a well-developed and full-fledged biofilm in the untreated mouse colon, as well as diverse types of bacteria adhering to its surface, which included rod-shaped and spherical bacilli. (Fig. 3b). However, in the control group, biofilm and bacterial adhesion on the colonic surface were rarely visible (Fig. 3c). Meanwhile, a thin biofilm was visible in the colon of the AI-2 group, and the bacteria adhering to the surface were spherical and 2.3 × 2.2 μm in size (Fig. 3d). Notably, the *LGG* + AI-2 group had considerably more biofilms and *LGG* on the surface of the colonic mucosa than in the *LGG* group (Fig. 3e, f). In addition, at the genus level (Fig. 3g), the mean relative abundance of *Lacticaseibacillus* in the *LGG* + AI-2 group was greater than that in the *LGG* group (*P* < 0.05). These results suggested that AI-2 might promote the formation of intestinal biofilms and the adhesion of *LGG* in the antibiotic-induced intestinal dysbiosis neonatal mouse model.

### The combination of AI-2 with *LGG* altered the expression several genes involved in intestinal barrier function

**Fig. 4:**
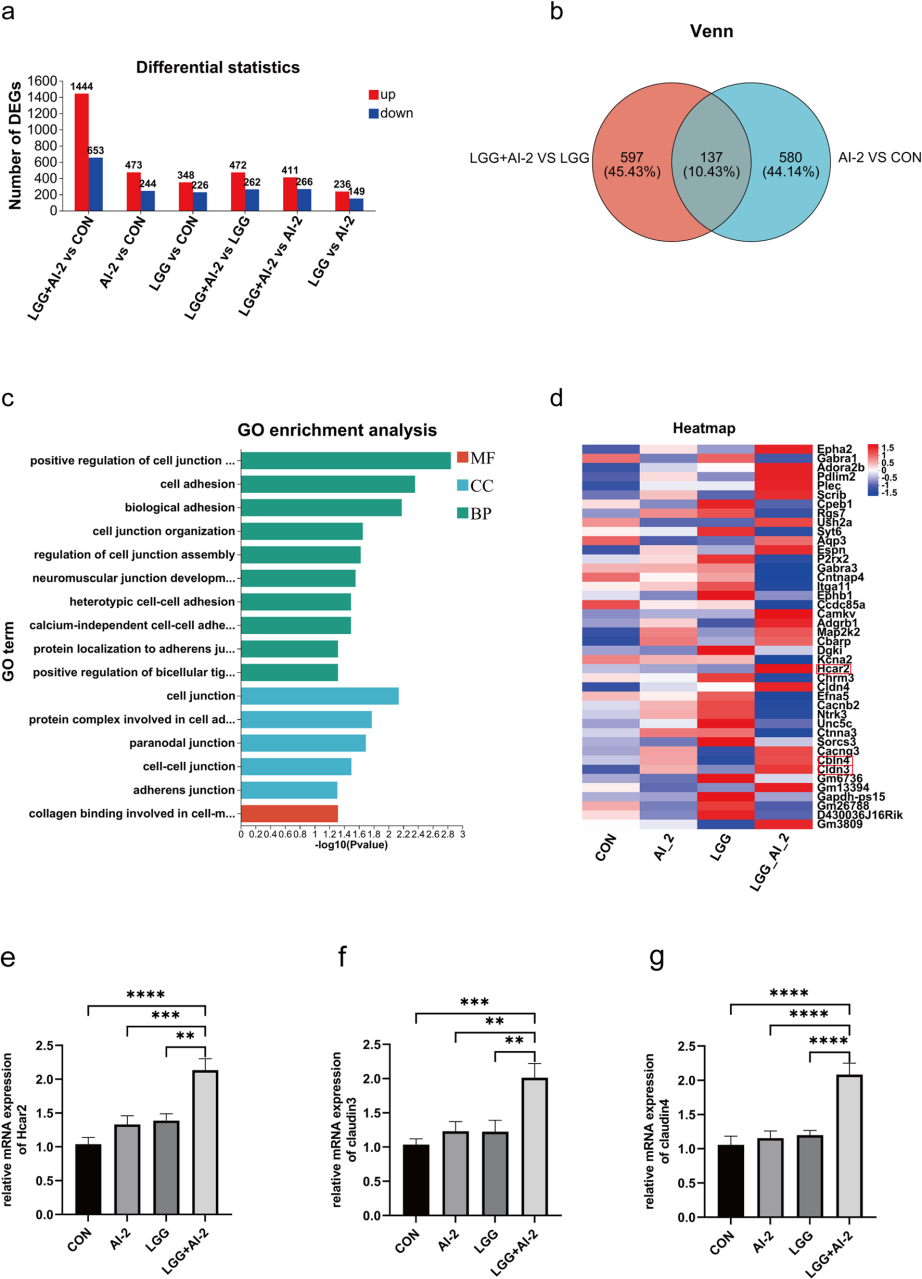
The differences in the intestinal tissue transcriptome in the four groups. **a** Numbers of differentially expressed genes (DEGs) among the control, AI-2, *LGG*, and *LGG*+AI-2 four groups. **b** Venn diagram analysis between the AI-2 *vs*. control groups and between the *LGG*+AI-2 *vs*. LGG groups. **c** GO_BP enrichment analysis, GO_MF enrichment analysis, and GO_CC enrichment analysis for DEGs except for the AI-2 *vs.* control groups between the *LGG*+AI-2 *vs. LGG* groups. **d** Heat map analysis of DEGs associated with the cell junction (n = 7 per group). **e**–**g** Comparison of *Hcar2*, *cldn3*, and *cldn4* mRNA expression levels among the four groups (n = 9 per group). DEGs were identified according to fold change≥1.5 and a P-value < 0.05 (DESeq2). Values are presented as the mean ± SEM. Significance was tested using one-way ANOVA. *P < 0.05, **P < 0.01, ***P < 0.001, ****P < 0.0001. BP, biological process; MF, molecular function; CC, cellular component

The above results suggested that AI-2 combined with *LGG* might facilitate *LGG* colonization and remodel the partially imbalanced intestinal flora in the antibiotic-induced intestinal dysbiosis neonatal mouse model. Therefore, intestinal tissue transcriptome analysis was performed to further investigate the molecular mechanism of AI-2 on the incidence of intestinal *LGG* colonization. Differentially expressed genes (DEGs) were identified using the criteria of *P-*value < 0.05 and Fold change > 1.5. First, we analyzed the number of DEGs in a two-by-two comparison between the control, AI-2, *LGG*, and *LGG* + AI-2 groups. As seen in Fig. 4a, compared with the control group, 653 genes were significantly downregulated and 1444 genes were significantly upregulated in the *LGG* + AI-2 group, 244 genes were significantly downregulated and 473 genes were significantly upregulated in the AI-2 group, and 226 genes were significantly downregulated and 348 genes were significantly upregulated in the *LGG* group. In the *LGG* + AI-2 group compared with the *LGG* group, 472 genes showed a significant upregulation, while 262 genes showed a significant downregulation. In the comparison between the *LGG* + AI-2 group and the AI-2 group, 411 genes were significantly upregulated and 266 genes were significantly downregulated. In the comparison between the *LGG* group and the AI-2 group, 236 genes were significantly upregulated, while 149 genes were significantly downregulated.

Then, we performed Venn diagram analysis of common and specific genes between the *LGG* + AI-2 group, the control group, and the *LGG* group. As seen in Fig. 4b, a total of 597 and 580 unique DEGs, as well as 137 common DEGs, were identified in the *LGG* + AI-2 *vs*. *LGG* and the AI-2 *vs*. control comparisons. To investigate the mechanism of AI-2's effects on *LGG* alone, GO analysis was performed on the 597 specific DEGs from the *LGG* + AI-2 *vs*. *LGG* comparison. As shown in Fig. 4c, the GO categories were enriched in biological process that were mostly related with cell junction, cell adhesion, biological adhesion, and tight junction assembly; similarly, in cellular component, the DEGs were primarily linked with cell junction, cellular adhesion, and adhesion junction; and in molecular function, they associated with cellular and matrix adhesion. Next, heat map analysis of cell junction-related genes was carried out (Fig. 4d), which showed that the expression levels of *Hcar2*, *Cldn3*, and *Cldn4* genes in the *LGG* + AI-2 group were higher than those in the control group, the AI-2 group, and the *LGG* group. Moreover, the results of qRT-PCR also confirmed that the expression levels of *Hcar2* (Fig. 4e, P < 0.0001, *P* < 0.001, and* P* < 0.01, respectively), *Cldn3* (Fig. 4f, P < 0.001, *P* < 0.01, and *P* < 0.01, respectively), and *Cldn4* (Fig. 4g, P < 0.0001) in the *LGG* + AI-2 group were substantially higher than those in other three groups. These data suggested that the combination of AI-2 with *LGG* promoted the expression of cell junction-related genes, which in turn improved intestinal barrier function in the antibiotic-induced intestinal dysbiosis neonatal mouse model.

### The combination of AI-2 with *LGG* enhanced protein expression levels associated with the intestinal barrier and repressed inflammatory cytokines in the antibiotic-induced intestinal dysbiosis neonatal mouse model

**Fig. 5:**
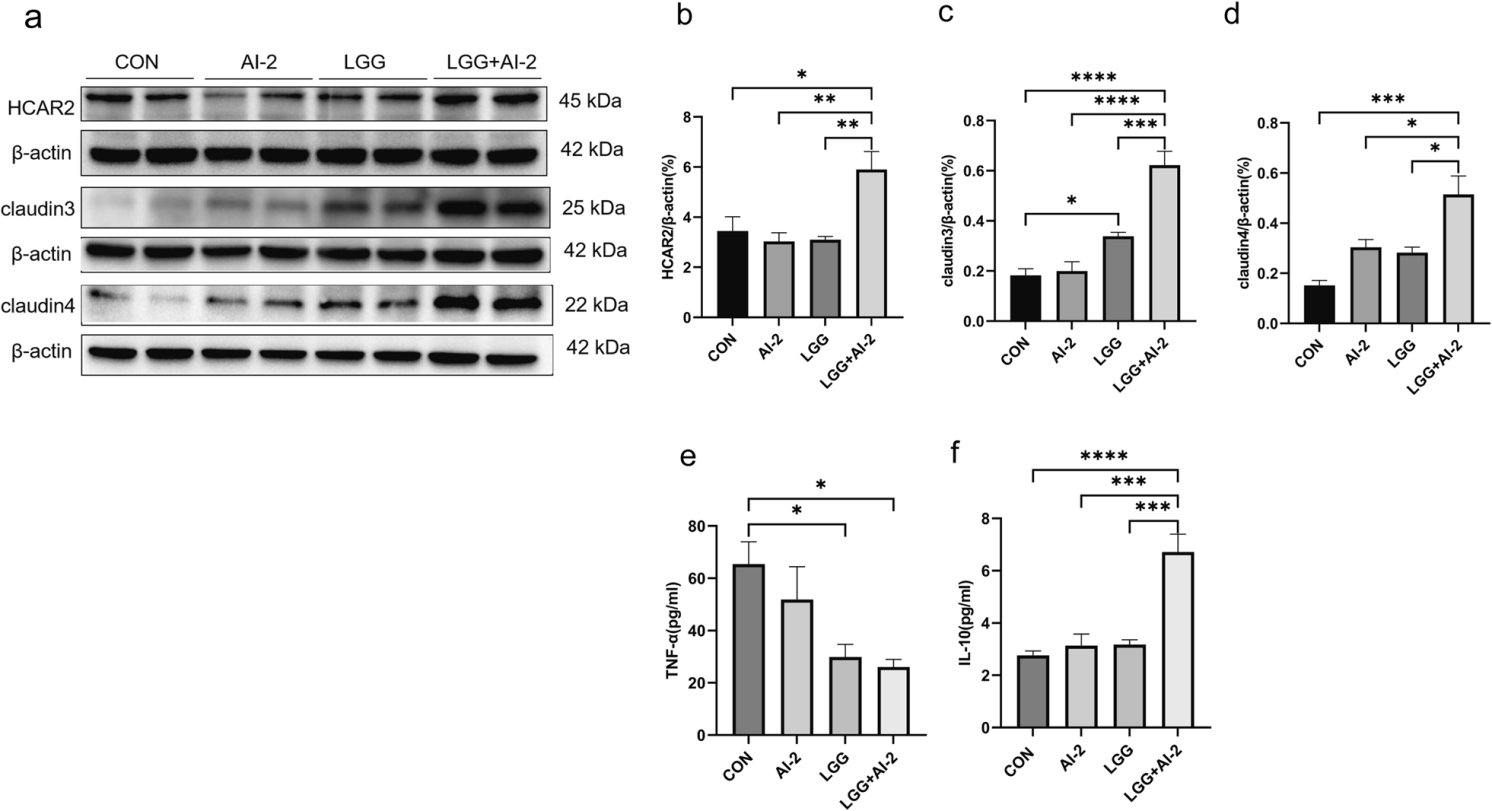
Changes in gut barrier-associated protein levels and inflammatory cytokine levels in the antibiotic-induced neonatal mouse model after treatment with AI-2 combined with *LGG*. **a** Western blotting analysis of Hcar2, claudin3, and claudin4 levels in the intestine among the four groups. **b**–**d** Quantitative analysis of Hcar2, claudin3, and claudin4 levels normalized to that of β-actin among the four groups (n = 4 per group). **e**, **f** ELISA-based comparison of TNF-α and IL-10 levels in the intestines among the four groups (n = 5 per group). Values are presented as the mean ± SEM. Significance was tested using one-way ANOVA. *P < 0.05, **P < 0.01, ***P < 0.001, ****P < 0.0001. TNF, tumor necrosis factor; IL-10, Interleukin 10

To investigate the effects of AI-2 treatment combined with *LGG* on barrier integrity and immune function in antibiotic-induced intestinal dysbiosis neonatal mouse model, tight junction proteins and inflammatory factors were detected using western blotting and ELISA, respectively. The Hcar2 protein level was considerably greater in the *LGG* + AI-2 group than in the control group (*P* < 0.05), AI-2 group (*P* < 0.01), and *LGG* group (*P* < 0.01). Similarly, the levels of claudin3 and claudin4 were significantly increased in the *LGG* + AI-2 group compared with those in the other three groups (*P* < 0.0001, *P* < 0.0001, and *P* < 0.001, respectively; *P* < 0.001, *P* < 0.05, and *P* < 0.05, respectively) (Fig. 5a–d). The ELISA results (Fig. 5e, f) revealed that the TNF-α level was significantly higher in the control group than in the *LGG* and *LGG* + AI-2 groups (both *P* < 0.05), whereas the IL-10 level was significantly higher in the *LGG* + AI-2 group than in the control, AI-2, and *LGG* groups (*P* < 0.0001, *P* < 0.001, and *P* < 0.01, respectively). These results suggested that AI-2 combined with *LGG* might affect the intestinal barrier and immune function in the antibiotic-induced intestinal dysbiosis neonatal mouse model.

### The combination of AI-2 with *LGG* increased the concentration of butyric acid in the gut of the antibiotic-induced intestinal dysbiosis neonatal mouse model

**Fig. 6:**
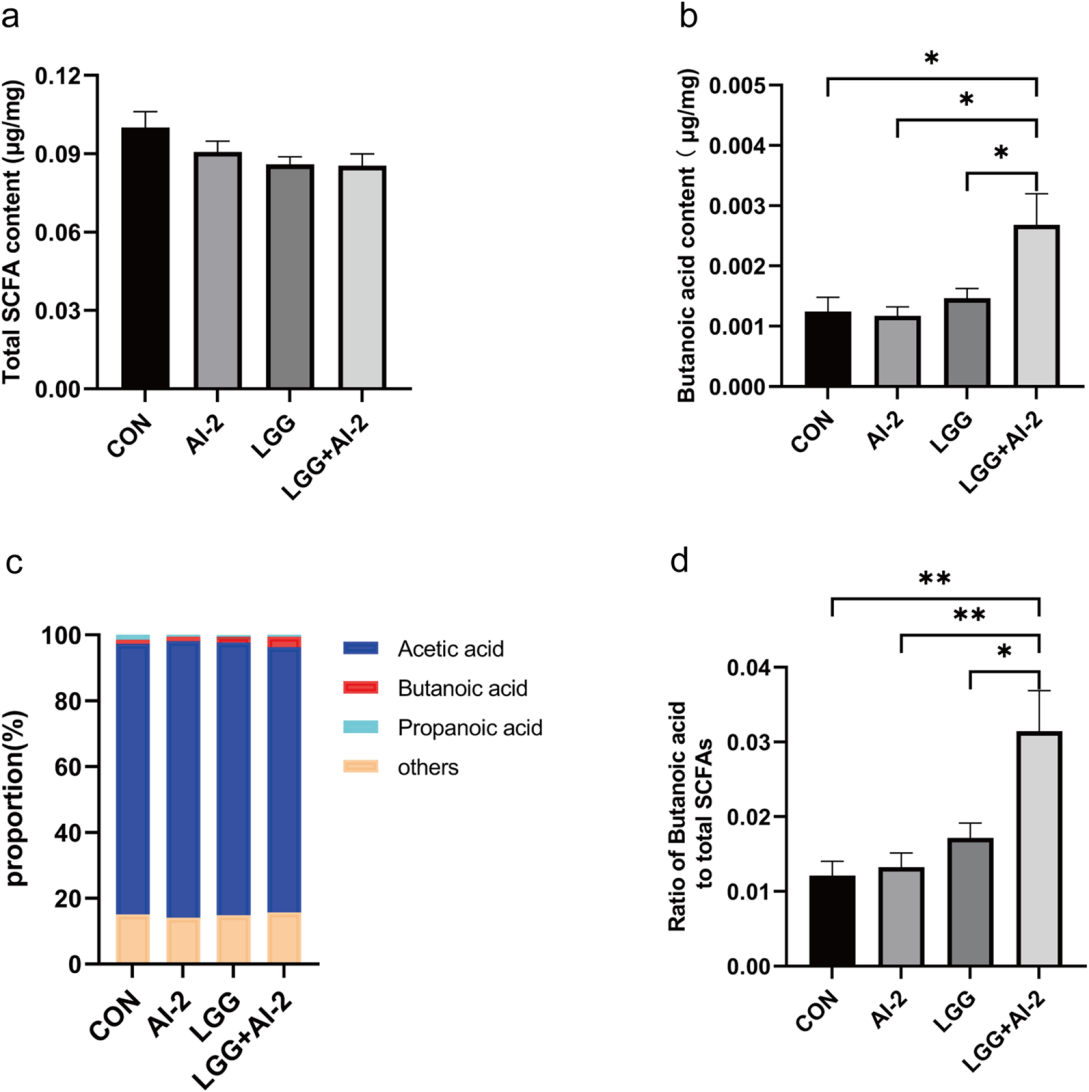
Levels of short-chain fatty acids in the gut of antibiotic-induced neonatal mice following the combination AI-2 with *LGG*. **a** Total short-chain fatty acids; **b** Butyric acid content; **c** The proportion of acetic acid, propionic acid, butyric acid, and others among total short-chain fatty acids. **d** The proportion of butyric acid in the four groups (n = 6 per group). Values are presented as the mean ± SEM. Significance was tested using one-way ANOVA. *P < 0.05, **P < 0.01

Acetic acid, propionic acid, and butyric acid are the main components of intestinal SCFAs, which are mostly generated by anaerobic bacteria utilizing undigested and absorbed carbohydrates for fermentation [41]. The overall amount of SCFAs was similar among the groups (Fig. 6a); however, the butyric acid content was considerably higher in the *LGG* + AI-2 group than in the control group, AI-2 group, or *LGG* group (Fig. 6b, P < 0.05). Next, we further analyzed the proportion of butyric acid among total SCFAs (Fig. 6c). The relative proportion of butyric acid was higher in the *LGG* + AI-2 group compared with that in the other three groups (Fig. 6d, P < 0.01, *P* < 0.01, and *P* < 0.05, respectively). Therefore, we concluded that AI-2 promotes butyric acid production by intestinal *LGG* in the antibiotic-induced intestinal dysbiosis neonatal mouse model.

### D-ribose reduced Hcar2, Claudin3 and Claudin4 expression levels in Caco-2 cells treated by AI-2 combined with *LGG*

**Fig. 7:**
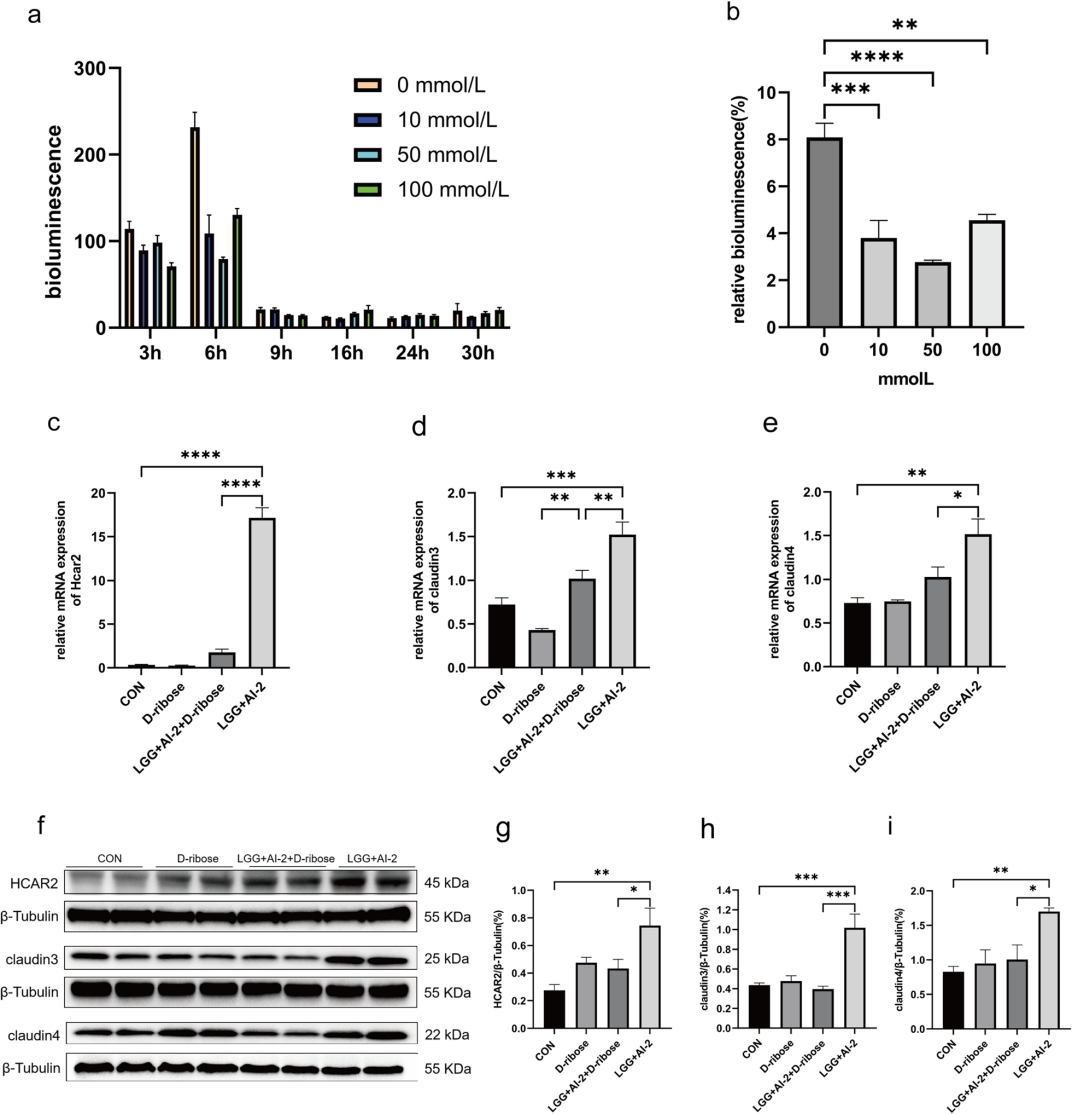
Changes in Hcar2, claudin3, and claudin4 expressions levels in the Caco-2 cells with AI-2+*LGG* treatment after D-ribose exposure. **a**, **b** Effects of different concentrations of D-ribose on the AI-2 activity of *LGG* at different times (n = 4 per group). **c**–**e** Comparison of *HCAR2*, *CLDN3*, and *CLDN4* mRNA expression levels among the groups (n = 3 per group). **f** Western blotting analysis of Hcar2, claudin3 and claudin4 protein levels among the groups. **g**–**i** Quantitative analysis of Hcar2, claudin3 and claudin4 protein levels normalized with that of the housekeeping gene among the four groups (n = 4 per group). Values are presented as the mean ± SEM. Significance was tested using one-way ANOVA. *P < 0.05, **P < 0.01, ***P <0.001, ****P < 0.0001

To identify whether D-ribose (an inhibitor of AI-2) inhibited AI-2 production by *LGG*, we applied different concentrations of D-ribose to LGG-infected Caco-2 cells at different time points and examined their relative biofluorescence values. The results showed that *LGG* had the highest biofluorescence value when it was incubated for 6 h. The biofluorescence value gradually decreased as the incubation time was prolonged (Fig. 7a), and 50 mmol/L D-ribose showed the strongest inhibition of AI-2 production by *LGG* after 6 h (Fig. 7b). Then, Caco-2 cells treated by AI-2 combined with *LGG* were co-cultured with 50 mmol/L D-ribose. The expression of *HCAR2*, *CLDN3*, and *CLDN4* mRNA in the *LGG* + AI-2 group was significantly higher than that in the control group, and when D-ribose was added, the mRNA expression levels of these genes decreased significantly (Fig. 7c–e). In accordance with prior findings, western blotting demonstrated a significant increase in Hcar2, Claudin3, and Claudin4 protein levels within Caco-2 cells of the *LGG* + AI-2 group compared with those in the control group. Subsequently, the addition of D-ribose resulted in a significant decrease in the levels of these proteins (Fig. 7f–i). These results supported the view that treatment with AI-2 combined with *LGG* enhanced the barrier function in Caco-2 cells, which could be inhibited by D-ribose.

### Effect of different concentrations of AI-2 on LGG adhesion to Caco-2 cells

While not substantially different from that at 500 nmol/L AI-2, the expression levels of the LGG adhesion genes spaC (Additional file 6: Fig S2a, P < 0.001, and P < 0.01, respectively), pilus444 (Additional file 6: Fig S2b, P < 0.001 and P < 0.01, respectively), and pili mRNA (Additional file 6: Fig S2c, P < 0.01) were considerably higher at 100 nmol/L AI-2 than those at 0 nmol/L and 10 nmol/L. And similarly, LGG adherence rate consistently revealed the same outcome (Additional file 6: Fig S2d, P < 0.01, and P < 0.05, respectively). The number of LGG strains adhering to Caco-2 cells observed by microscopy was also significantly higher at 100 nmol/L AI-2 concentration than at 0 nmol/L, 10 nmol/L and 500 nmol/L, as shown in Additional file 6: Fig S2e.

## Discussion

The mechanisms underlying the potential influence of AI-2 on intestinal *LGG* have not been clarified in an antibiotic-induced intestinal dysbiosis neonatal mouse model. The present study revealed that exogenous AI-2 promoted the colonization of *LGG* and increased the concentration of butyric acid to ameliorate the deteriorated intestinal mucosal barrier function in mice with antibiotic-induced intestinal dysbiosis.

The important role of LuxS/AI-2 in regulating bacterial colonization and biofilm formation has been widely studied. Autoinducer-2 (AI-2) is a signal molecule produced by the *LuxS* gene in many bacterial species and enables specific intraspecies communication [42], which regulates bacterial community behavior in a density-dependent manner [43]. Studies have shown that antibiotics severely affect the gut microbiota [44], leading to decreased intestinal AI-2 activity [19]. Nevertheless, in the current study, we found that the combination of AI-2 with *LGG* significantly increased the concentration of AI-2 in the intestines of mice treated with antibiotics compared with the same mice only supplemented with *LGG* or AI-2. This suggested that exogenous AI-2 interacts with *LGG*, resulting in increased concentrations of AI-2 in the gut.

Biofilms are intricate structures formed by the aggregation of microorganisms, including bacteria, fungi, or other microbial communities [45]. The formation of biofilms by probiotics in the gut can prolong bacterial residence time and facilitate nutrient exchange between the host and the microbiota [46]. Moreover, the biofilm formed by probiotics establishes a physical barrier, which contributes to maintain the abundance and functionality of beneficial microbiota in the gut while reducing the proliferation of harmful microorganisms [47]. Therefore, the effective colonization of probiotics in intestines is crucial for their probiotic function, which can improve the survival and colonization of probiotics in the gut [48]. It has been reported that AI-2 promotes the colonization and synergistic effects of probiotics within the biofilm, thereby enhancing the stability and functionality of the gut microbiota [49–51]. Importantly, during the early stages of life, especially following antibiotic use, the gut biofilm is not fully mature, and the non-biofilm states of microbial communities are highly vulnerable to external factors [52]. Our research revealed that the combination of AI-2 with LGG promotes the colonization of LGG and biofilm formation in the gut, significantly increasing the proportion of Firmicutes, while competitively decreasing the fraction of Proteobacteria. Another study has shown that cocktails of Lactobacillus and fructooligosaccharide effectively restore the composition of the gut microbiota in cefixime-induced dysfunction in gut microbiota, which increase the abundance of Firmicutes and reduce Bacteroidetes, Proteobacteria [53]. Likewise, AI-2 dramatically altered the composition of the gut microbiota in antibiotic-treated mice, favoring Firmicutes and impeding Bacteroides [54]. In our study, the combination of AI-2 with *LGG* significantly increased the intestinal AI-2 concentration, resulting in a change in the intestinal environment that allows *LGG* to better colonize the gut [55], which partially reshaped the microbiota structure in neonatal mouse model of the antibiotic-induced intestinal dysbiosis. Meanwhile, the cell adhesion assay further clarified that the *LGG* adhesion ability was significantly increased by the addition of AI-2, suggesting the importance of AI-2 in biofilm formation and adhesion of *LGG*. A *luxS* mutant of *LGG* showed reduced AI-2 activity, biofilm formation, and adhesion, and the addition of AI-2 partially rescued these deficiencies in the mutant strain [23]. Hence, as a bacterial signaling molecule, AI-2 interacts with *LGG*, possibly through alteration of specific signaling pathways and metabolites. However, the underling mechanism still requires further investigation.

To examine the effect of AI-2 on *LGG* in antibiotic-induced intestinal disorders, we performed intestinal tissue transcriptome sequencing. The results indicated that the effect of AI-2 combined with *LGG* on the intestines mainly involves genes related to cell junctions and barrier function. The three significantly altered genes we identified in transcriptome sequencing were *Hcar2*, *Cldn3*, and *Cldn4*, whose encoded proteins are all associated with intestinal barrier and immune function [55, 56]. Claudin3 and Claudin4 are important tight junction proteins, which act as a barrier to prevent harmful substances from entering the bloodstream [57]. As reported, broad-spectrum antibiotics can decrease expression of tight junction proteins and oral supplementation with *LGG* minimizes these losses [58]. The present study showed that compared with supplementation with *LGG* or AI-2 alone, the combination of AI-2 with *LGG* significantly enhanced the levels of Claudin3 and Claudin4 in vivo*.* More importantly, it was further confirmed that treatment with D-ribose, an inhibitor of AI-2, dramatically reduced the increased levels of Claudin3 and Claudin4 in Caco-2 cells induced by AI-2 plus *LGG*. Thus, the combination of AI-2 and *LGG* might contribute to the reinforcement of the intestinal barrier function in a synergistic manner. However, further research is needed to determine the mechanism of this combined effect on the intestinal barrier function.

Butyric acid, known as a "beneficial" SCFA, exerts multifaceted biological roles, encompassing anti-inflammatory, antioxidant, and immunomodulatory effects [59–61], which play an important role in maintaining intestinal health [62]. Hcar2 (also known as GPR109a) is a G protein-coupled receptor expressed in the colonic epithelium, which is primarily activated by butyric acid [63, 64]. It is widely recognized that antibiotics lead to a distinct alteration in gut microbiota diversity, characterized by a significant decrease in the prevalence of butyrate-producing bacteria, and consequently, a reduction in intestinal butyric acid levels [65]. It has been proven that broad-spectrum antibiotics significantly decreased the concentrations of SCFA in the faeces of mice and decreased expression of butyrate transporter and receptor. However, oral supplementation with LGG and/or tributyrin effectively mitigated these losses [53, 58]. Notably, the administration of *LGG* also augmented the production of butyric acid in cecal contents, which attenuated deoxynivalenol (DON)-induced inflammation and impairment of the intestinal barrier function [66]. These effects collectively contribute to enhancing the function of the intestinal barrier. In this study, the combination of AI-2 with *LGG* significantly increased the butyric acid concentration and the Hcar2 level in the intestines compared with those in the *LGG* or AI-2 supplementation only. More importantly, in Caco-2 cell experiments, we confirmed that AI-2 combined with *LGG* increased Hcar2 levels (and by extension would improve the barrier function of intestinal epithelial cells in situ), which was counteracted by D-ribose. Thus, AI-2 may allow for better colonization of *LGG* in the gut, which in turn would increase intestinal butyric acid production to promote host health. In addition to activating Hcar2, butyric acid acts as an inhibitor of histone deacetylase (HDAC), which reduces histone acetylation and stimulates gene expression in host cells [67]. Recent studies have reported that butyric acid improves the expression of tight junction proteins and intestinal barrier function by inhibiting histone acetylation [33, 68]. However, more studies are required to confirm whether the combination of AI-2 with *LGG* modulates the intestinal barrier function by inhibiting HDAC via butyric acid.

TNF-α is a potent driver of inflammation, playing a pivotal role in initiating and amplifying inflammatory processes that worsen various inflammatory diseases. In contrast, IL-10 serves as an anti-inflammatory factor, known for its ability to mitigate inflammation and promote the recovery of damaged tissues [69]. Previous research has demonstrated that the exogenous administration of AI-2 contributes to a reduction in intestinal TNF-α and an increase in IL-10, resulting in the attenuation of the inflammatory responses in mice with necrotizing enterocolitis (NEC) [20]^.^ Similarly, we found that AI-2 combined with *LGG* significantly reduced TNF-α levels and significantly increased IL-10 levels, underlining that AI-2 has an important regulatory role in intestinal immunity. However, *LGG* supplementation was ineffective in alleviating the inflammatory response of the ileal pouch [70]. Therefore, the combination of AI-2 with *LGG* might have a more substantial effect on intestinal immune function. However, it is important to note that precise mechanisms still require further investigation and clarification.

The present study has certain limitations that need to be acknowledged. Firstly, further research is required to comprehensively understand the specific mechanism by which AI-2 regulates the colonization of LGG in antibiotic-induced intestinal dysbiosis. Secondly, additional studies are needed to confirm whether the combination of AI-2 with LGG modulates the intestinal barrier function by inhibiting HDAC via butyric acid. Lastly, we predominantly focused on the P-value in statistical analyses, and we acknowledge the importance of incorporating effect size in future research [38, 71].

## Conclusions

This study revealed that the combination of AI-2 with *LGG* promoted the colonization of *LGG* and intestinal biofilm formation, and partially reshaped the structure of the microbiota of antibiotic-induced intestinal dysbiosis by enhancing *Firmicutes* and *Lacticaseibacillus spp.* and reducing *Proteobacteria*. In addition, AI-2 combined with *LGG* increased the concentration of butyric acid, promoted the levels of Claudin3, Claudin4, and Hcar2, and improved intestinal immune barrier function. Finally, in vitro experiments confirmed that D-ribose could reverse the increases in Claudin3, Claudin4 and Hcar2 levels in Caco-2 cells treated with the combination of AI-2 with *LGG*. These findings support the potential of AI-2 and *LGG* in regulating the gut microbiota and host intestinal health.

### Supplementary Information

**Additional file 1:** The community richness indicators among the four groups.

**Additional file 2:** The relative abundance on Phylum level among the four groups.

**Additional file 3:** The relative abundance on Family level among the four groups.

**Additional file 4:** The relative abundance on Genus level among the four groups.

**Additional file 5:** **Figure S1.** Bray-Curtis(a–c) and Weighted UniFrac(d–f) of Principal Co-ordinates Analysis (PCoA) at the Phylum, Class, and Order levels in antibiotic-induced intestinal disorders.

**Additional file 6:** **Figure S2.** Effects of different concentrations of AI-2 on LGG adhesion to Caco-2 cells.

## Data Availability

The data used in the study analyses can be made available by the corresponding author on reasonable request.

## Abbreviations

*LGG*: *Lactobacillus rhamnosus GG*
AI-2: Autoinducer-2
NEC: Necrotizing enterocolitis
HE: Hematoxylin and eosin
CFU: Colony forming units
MRS: De Man, Rogosa, and Sharpe
DPD: S-4,5-dihydroxypentane-2,3-dione
PBS: Phosphate-buffered saline
SEM: Scanning electron microscopy
RIPA: Radio immunoprecipitation assay lysis bufer
PMSF: Phenylmethylsulfonyl fluoride
SDS: Sodium dodecyl sulfate
PVDF: Polyvinylidene difuoride
ECL: Enhanced chemiluminescence
DMEM: Dulbecco’s modified Eagle’s medium
Hcar2: Hydroxycarboxylic acid receptor 2
TNF-α: Tumor necrosis factor-α
IL-10: Interleukin-10
DEGs: Differentially expressed genes
GO: Gene Ontology
BP: Biological process
MF: Molecular function
CC: Cellular component
PCoA: Principal Co-ordinates Analysis
