# Supplementary material for: Autoinducer-2 promotes the colonization of Lactobacillus rhamnosus GG to improve the intestinal barrier function in a neonatal mouse model of antibiotic-induced intestinal dysbiosis
Source: J Transl Med. 2024 Feb 18;22:177. doi: 10.1186/s12967-024-04991-5 (PMC10874557; doi:10.1186/s12967-024-04991-5)
Supplement: Supplementary file 5 — Additional file 5: Figure S1. Bray-Curtis(a–c) and Weighted UniFrac(d–f) of Principal Co-ordinates Analysis (PCoA) at the Phylum, Class, and Order levels in antibiotic-induced intestinal disorders. [file 12967_2024_4991_MOESM5_ESM.doc]

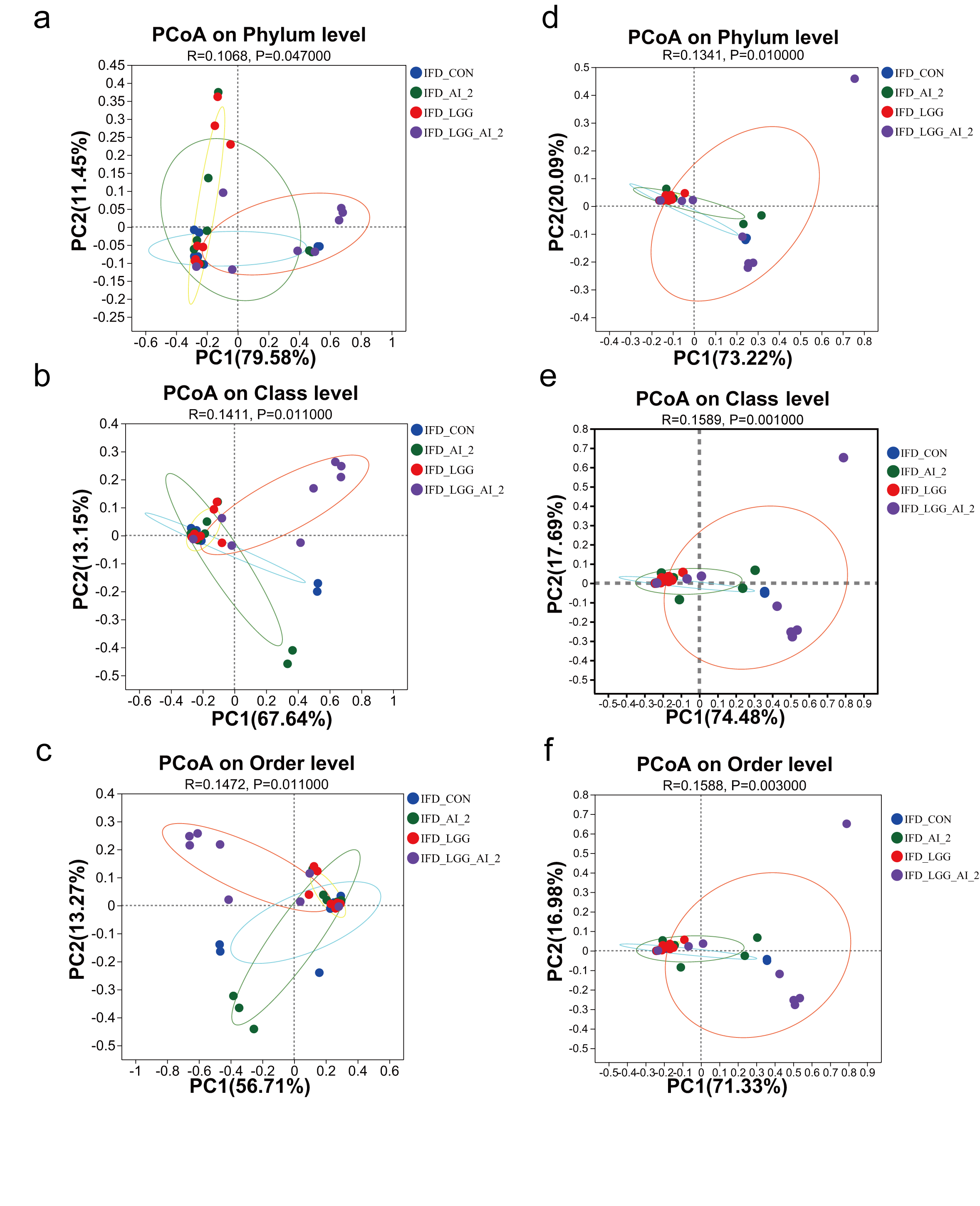


**Supplementary figure.1** Bray-Curtis(a-c) and Weighted UniFrac(d-f) of Principal Co-ordinates Analysis(PCoA) at the Phylum, Class, and Order levels in antibiotic-induced intestinal disorders.
