# Supplementary material for: Autoinducer-2 promotes the colonization of Lactobacillus rhamnosus GG to improve the intestinal barrier function in a neonatal mouse model of antibiotic-induced intestinal dysbiosis
Source: J Transl Med. 2024 Feb 18;22:177. doi: 10.1186/s12967-024-04991-5 (PMC10874557; doi:10.1186/s12967-024-04991-5)
Supplement: Supplementary file 6 — Additional file 6: Figure S2. Effects of different concentrations of AI-2 on LGG adhesion to Caco-2 cells. [file 12967_2024_4991_MOESM6_ESM.doc]

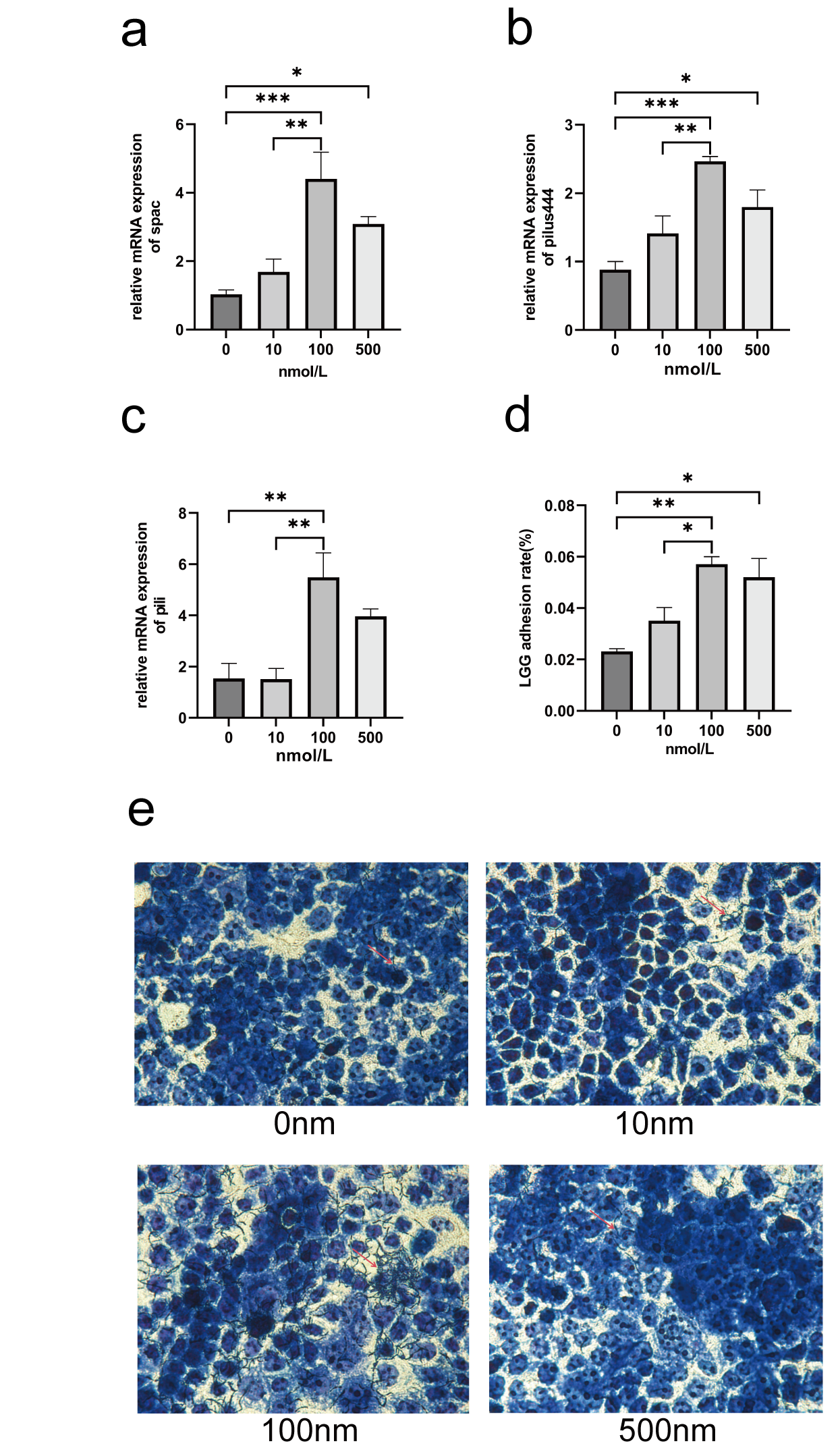


**Supplementary figure.2** Effects of different concentrations of AI-2(nmol/L) on LGG adhesion to Caco-2 cells. (a-c) Comparison of spaC, pilus444, pili mRNA expressions levels among the groups (n=5 per group). (d) Adhesion rate of LGG strains to Caco-2 cells in different concentrations of AI-2(n=3 per group). (e) Microscopic observation of LGG strains attachment on Caco-2 cells in different concentrations of AI-2. Three fields of vision were randomly selected for each slide under oil microscope (n=3 per group).
